# Supplementary material for: Mechanisms governing the pioneering and redistribution capabilities of the non-classical pioneer PU.1
Source: Nat Commun. 2020 Jan 21;11:402. doi: 10.1038/s41467-019-13960-2 (PMC6972792; doi:10.1038/s41467-019-13960-2)
Supplement: Supplementary file 7 — Source data [file 41467_2019_13960_MOESM7_ESM.zip › Source_Data/FigureS2/FigureS2B_MotifScanOutput/KmeansCluster2/homerResults/motif4.similar.html]

motif4

## Information for motif4

C
T
G
A
T
A
C
G
C
G
T
A
C
T
A
G
T
C
A
G
T
G
C
A
C
G
T
A
T
A
C
G
C
G
T
A
C
A
T
G
C
T
A
G
T
G
C
A
C
G
T
A
C
T
A
G
  
Reverse Opposite:  

G
A
T
C
G
C
A
T
A
C
G
T
G
A
T
C
G
T
A
C
G
C
A
T
A
T
G
C
G
C
A
T
A
C
G
T
A
G
T
C
G
A
T
C
G
A
C
T
A
T
G
C
G
A
C
T
  

|  |  |
| --- | --- |
| p-value: | 1e-426 |
| log p-value: | -9.824e+02 |
| Information Content per bp: | 1.639 |
| Number of Target Sequences with motif | 1694.0 |
| Percentage of Target Sequences with motif | 33.72% |
| Number of Background Sequences with motif | 3707.3 |
| Percentage of Background Sequences with motif | 9.12% |
| Average Position of motif in Targets | 104.9 +/- 48.1bp |
| Average Position of motif in Background | 101.1 +/- 72.3bp |
| Strand Bias (log2 ratio + to - strand density) | -0.2 |
| Multiplicity (# of sites on avg that occur together) | 1.01 |
| Motif File: | file (matrix) reverse opposite |

### Similar de novo motifs found

|  |  |  |  |  |  |  |  |
| --- | --- | --- | --- | --- | --- | --- | --- |
| Rank | Match Score | Redundant Motif | P-value | log P-value | % of Targets | % of Background | Motif file |
| 1 | 0.882 | A G T C G A C T A G C T G A T C G A T C G A C T A G T C G A C T A G C T A G T C G A T C G A C T A G T C | 1e-357 | -823.114409 | 29.58% | 8.15% | motif file (matrix) |
| 2 | 0.891 | C G T A T A C G C G T A C A T G A T C G G C T A C G T A A T C G C G T A A C T G T C A G C T G A C G T A | 1e-204 | -470.356101 | 10.21% | 1.41% | motif file (matrix) |
